# Food anticipatory activity on a calorie-restricted diet is independent of Sirt1

**DOI:** 10.1371/journal.pone.0199586

**Published:** 2018-06-25

**Authors:** Dina R. Assali, Cynthia T. Hsu, Keith M. Gunapala, Antonio Aguayo, Michael McBurney, Andrew D. Steele

**Affiliations:** 1 Department of Biological Sciences, California State Polytechnic University Pomona, Pomona, CA, United States of America; 2 Division of Biology, California Institute of Technology, Pasadena, CA, United States of America; 3 Department of Medicine, University of Ottawa, Ottawa, ON, Canada; Kent State University, UNITED STATES

## Abstract

A number of studies have demonstrated that the Sirtuin family member, Sirt1, is a key integrator of growth, metabolism, and lifespan. Sirt1 directly interacts with and deacetylates key regulators of the circadian clock, positioning it to be an important link between feeding and circadian rhythms. In fact, one study suggests that Sirt1 is necessary for behavioral anticipation of limited daily food availability, a circadian process termed food anticipatory activity (FAA). In their study, mice overexpressing Sirt1 had enhanced FAA, while mice lacking Sirt1 had little to no FAA. Based on the supposition that Sirt1 was indeed required for FAA, we sought to use Sirt1 deletion to map the neural circuitry responsible for FAA. We began by inactivating Sirt1 using the cell-type specific Cre-driver lines proopiomelanocortin, but after observing no effect on body weight loss or FAA we then moved on to more broadly neuronal Cre drivers Ca2+/calmodulin-dependent protein kinase II and nestin. As neither of these neuronal deletions of Sirt1 had impaired FAA, we then tested 1) a broad postnatal tamoxifen-inducible deletion, 2) a complete, developmental knockout of Sirt1, and 3) a gene replacement, catalytically inactive, form of Sirt1; but all of these mice had FAA similar to controls. Therefore, our findings suggest that FAA is completely independent of Sirt1.

## Introduction

The ability to anticipate resource availability plays a crucial role in survival. When rodents are fed a limited amount at the same time daily, they demonstrate increased locomotor activity and body temperature prior to the regularly scheduled feeding time [1], suggesting that food can serve as a circadian regulator. There is a rich history of attempts to study the neural basis of food anticipatory activity (FAA); both brain lesions and genetic manipulations have yielded inconsistent results and there is no widespread agreement on which neurons mediate FAA [2–7]. Given the difficulty in finding the neural substrate(s) of FAA, it has been suggested that there is no discreetly localized food entrainable oscillator (FEO) that can be inhibited through lesions or single gene deletion [2, 8]. That being said, one study implicates the nicotinamide adenine dinucleotide (NAD+)-dependent deacetylase, Sirt1, as being required for FAA [9]. Since this study was done with widespread transgenic overexpression and complete deletion of Sirt1, using more refined deletion methods presents an excellent opportunity to map the circuitry behind FAA.

Indeed, Sirt1 is known to physically interact with key components of the core circadian clock and is also regulated by metabolism, which is highly circadian in nature [10,11]. The circadian clock is encoded by a transcription-translation feedback loop that synchronizes behavior and metabolism with the light-dark cycle and feeding. The rate-limiting enzyme in mammalian nicotinamide adenine dinucleotide (NAD+) biosynthesis, nicotinamide phosphoribosyltransferase (NAMPT), and levels of NAD+ display circadian oscillations that are regulated by the core clock machinery in mice. Inhibition of NAMPT promotes oscillation of the clock gene Per2 by releasing CLOCK:BMAL1 from suppression by Sirt1. In turn, the circadian transcription factor CLOCK binds to and up-regulates NAMPT, thus completing a feedback loop involving NAMPT/NAD+ and Sirt1/CLOCK:BMAL1 [12]. These include, but are not limited to, histones, BMAL1, and PER2 [13–15]. Sirt1 also deacetylates peroxisome proliferators activated receptor (PPAR)-γ and the peroxisome proliferators activated receptor co-activator (PGC-1α), which control fatty acid mobilization and oxidation [16, 17], and coordinate gluconeogenesis [18]. Thus, a number of experiments have positioned Sirt1 as an essential conduit between metabolism and circadian machinery [19].

The role of Sirt1 in the hypothalamus is controversial, as both deleting Sirt1 [20] and enhancing its activity both locally [21] as well as globally [22] leads to hyperphagia. Deleting Sirt1 in pro-opiomelanocortin (POMC) neurons is sufficient to reduce energy expenditure in mice fed on a hypercaloric diet [23]. Pan-neuronal deletion of Sirt1 eliminates CR-induced hyperactivity, reduces growth hormone secretion, and glucose intolerance in older mice [24]. Sirt1 global knockouts lack the increased activity and lifespan typical of mice on CR [25, 26]. Finally, Satoh and colleagues (2010) demonstrate that transgenically increasing levels of Sirt1 leads to stronger activation of metabolic centers in the hypothalamus along with increased physical activity when calorie intake is restricted [9]. Correspondingly, knocking out Sirt1 globally decreased hypothalamic neuronal activation and almost completely abrogated FAA, essentially demonstrating that Sirt1 is the missing link between circadian activity cycles and scheduled feeding. Inspired by these results, we attempted to delete Sirt1 in a cell-type-specific manner using the Cre-Lox system in mice to finely map the neuronal circuitry required for FAA.

## Results

**Fig 1:**
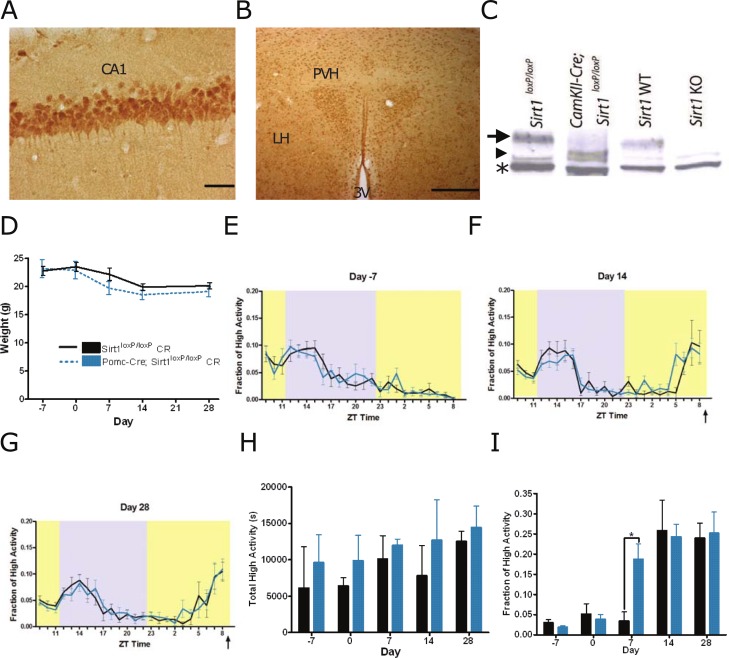
Sirt1 expression, deletion, and testing FAA in mice lacking Sirt1 in Pomc neurons. Immunostaining for Sirt1 in the adult mouse brain reveals a broad neuronal expression pattern: (A) CA1 region of the hippocampus (scale bar indicates 100 microns) and (B) low magnification image of the dorsal hypothalamus (scale bar indicates 500 microns), showing robust Sirt1 expression in the paraventricular nucleus. (C) Western blot showing the decreased molecular weight of Sirt1 in homogenates of cortex of *CamKII-Cre; Sirt1*^*loxP/loxP*^ mice (lane 2) indicated by an arrowhead. Lane 1 shows full length Sirt1 protein indicated by an arrow in *Sirt1*^*loxP/loxP*^ control cortex. Lanes 3 and 4 are from the cortex of a *Sirt1* WT and *Sirt1* KO mice. Note that there is a cross reacting protein of smaller molecular weight than the exon 4-deleted Sirt1 indicated by an asterisk. (D) Body weights of *Pomc-Cre; Sirt1*^*loxP/loxP*^ and *Sirt1*^*loxP/loxP*^ littermate controls. Mice were fed AL diets on Day -7. CR began on Day 0. Waveform of high activity behaviors at (E) day -7 (n = 6 WT, n = 8 KO), prior to initiating timed CR, (F) day 14 (n = 6 WT, n = 8 KO) of CR, (G) day 28 (n = 6 WT, n = 8 KO) of CR. (H) Total high activity (walking, hanging, jumping, and rearing) observed during the duration of the 23.5h to 24h video across the duration of the experiment. (I) High activity observed during the three hours preceding scheduled feeding divided by the total seconds of total high activity is depicted as fraction of high activity to represent FAA in mice. Data are shown as means +/- SEMS. Feeding time is indicated by an arrow. Lights on are indicated by yellow coloration while lights off time is indicated by grayed area. For body weight, statistical significance was determined using an unpaired t-test; for behavioral data, statistical significance was determined using the Mann-Whitney Test. * denotes p<0.05.

Consistent with the findings of others [9, 23, 27], we observed widespread neuronal expression of Sirt1 using immunohistochemistry in adult mouse brain fed on an *ad-libitum diet*. Sirt1 showed robust expression in regions such as the hippocampus (Fig 1A) and regions important in metabolism, including the paraventricular nucleus of the hypothalamus (Fig 1B).

Our initial approach was to determine if targeted deletion of Sirt1 in selected populations of neurons could suppress FAA in pilot studies (Table 1). We made use of the Cre-lox system to inactivate Sirt1 by deleting its fourth exon, the catalytic domain, in several brain regions *Sirt1*^*loxP/loxP*^ [28]. Note that the deletion of the fourth exon of Sirt1 mimics the phenotypes observed by the complete loss of Sirt1 protein in the initial knockout mouse study [28, 29]. We used Western blotting to verify deletion of exon 4 of Sirt1, noting a decrease in the size of Sirt1 protein in forebrain homogenates from *CamKII-Cre; Sirt1*^*loxP/loxP*^ mice (Fig 1C) and brain lysates from a complete knockout of Sirt1 to verify the specificity of the Sirt1 antibody [29]. The nearly complete downward shift of the molecular weight of Sirt1 in *CamKII-Cre; Sirt1*^*loxP/loxP*^ brain lysate suggests a high efficiency of deletion of the 4th exon, consistent with other studies using this conditional allele [28].

**Table 1:** Mouse strains used in this study.

Strain	Sirt1 allele	Area of expression/deletion	Reference
Sirt1^loxP/loxP^	Floxed Exon 4	Conditional allele	[28]
Sirt1 KO	Null allele	Global	[29]
Sirt1 Y/Y Mutant	No catalytic activity	Global	[36]
Tyrosine hydroxylase-Cre	Floxed Exon 4	Dopamine, norepinephrine, and epinephrine producing cells	[32]
Pomc-Cre	Floxed Exon 4	Pomc neurons, mainly arcuate nucleus and nucleus tractus solitarius	[30]
CAGG-Cre^Tm^	Floxed Exon 4	Global, 10 weeks of age at tamoxifen injection	[35]
Nestin-Cre	Floxed Exon 4	Neuronal lineages	[34]
CamKII-alpha-Cre	Floxed Exon 4	Forebrain	[33]

Deleting Sirt1 in POMC neurons using *Pomc-Cre* mice [30] did not cause any significant body weight loss observed in *Pomc-Cre*; *Sirt1*^*loxP/loxP*^ mice and the *Sirt1*^*loxP/loxP*^ littermate controls on a timed 60% calorie restricted (CR) diet (Fig 1D and S1 Dataset). *Pomc-Cre*; *Sirt1*^*loxP/loxP*^ mice had an average daily food intake of 5.6 grams (+/- 1.1g), while the *Sirt1*^*loxP/loxP*^ controls had an intake of 5.1 grams (+/-1.2g) (P = 0.43, t test). Next, we studied the behavioral response to timed CR in these mice, measuring the amount of high activity, or summation of walking, rearing, jumping, and hanging, in videos of home cage behavior using computer vision software [31] weekly. Starting 7 days prior to initiating CR, “Day -7”, we observed similar normalized activity waveforms (the high activity in each hourly bin divided by the total high activity) between *Pomc-Cre*; *Sirt1*^*loxP/loxP*^ mice and *Sirt1*^*loxP/loxP*^ controls (Fig 1E). After 14 and 28 days of timed CR, both groups of mice show a strong peak of activity preceding scheduled mealtime at ZT9 (Fig 1F and 1G). There were similar levels of total high activity (in seconds) between *Pomc-Cre*; *Sirt1*^*loxP/loxP*^ mice and *Sirt1*^*loxP/loxP*^ controls (Fig 1H). To quantify the amount of FAA, we divided the activity in the 3 hours (h) prior to scheduled mealtime by the total high activity for the 24 hour recording to express the ‘fraction of high activity’, our working definition for FAA. For example, on the first day of timed CR (“day 0”), both *Pomc-Cre*; *Sirt1*^*loxP/loxP*^ and *Sirt1*^*loxP/loxP*^ mice expend only 4–5% of their total high activity behaviors in the 3h preceding scheduled mealtime (Fig 1I). We noted that *Pomc-Cre*; *Sirt1*^*loxP/loxP*^ developed FAA more quickly than control mice as they demonstrated a statistically significant increase in FAA after 7 days of CR (P = 0.01, Mann-Whitney, Fig 1F), but at all time points beyond Day 7 there were no significant differences in pre-meal activity.

**Fig 2:**
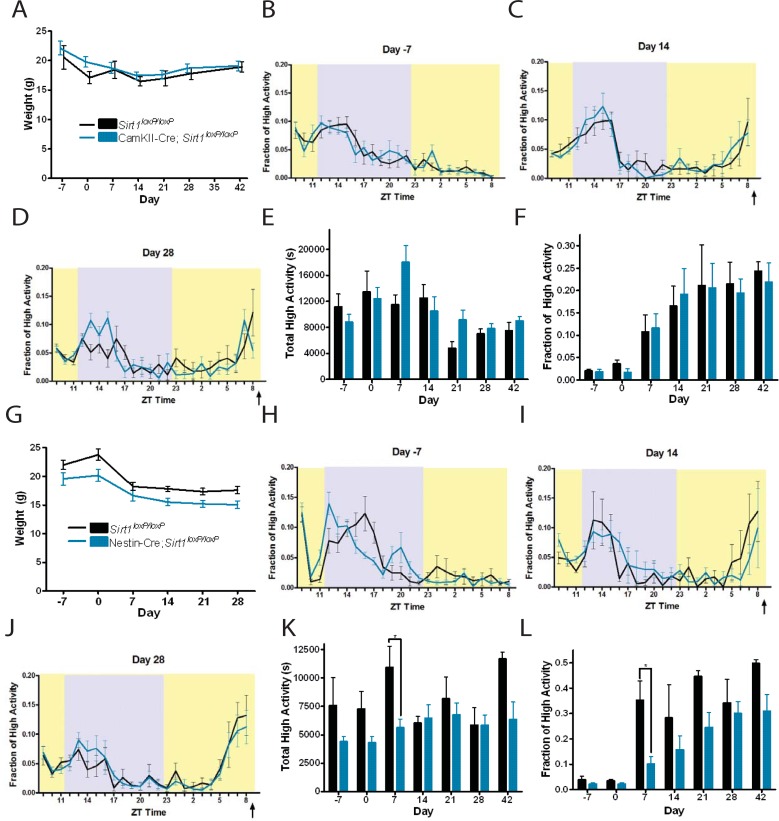
Testing FAA in forebrain and pan neuronal deletion mutants of Sirt1. (A) Mean body weights for *CamKII-Cre; Sirt1*^*loxP/loxP*^ and *Sirt1*^*loxP/loxP*^ littermate controls. CR diets began on Day 0. Mean high activity data for home cage behaviors at (B) Day -7 (n = 8 WT and n = 9 for KO), (C) Day 14 of CR (n = 6 WT and n = 9 KO), and (D) Day 28 (n = 7 WT and n = 8 KO) of CR are shown. (E) Mean total high activity, measured in seconds, over the entire 23.5h-24h video recording for *Sirt1*^*loxP/loxP*^ controls and CamKII-Cre;*Sirt1*^*loxP/loxP*^ (F) Mean fraction of normalized high activity in the 3h prior to mealtime; there were no differences between groups on all days. (G) Mean body weight for *Nestin-Cre; Sirt1*^*loxP/loxP*^ and *Sirt1*^*loxP/loxP*^ littermate controls. Mean high activity data for home cage behaviors at (H) Day -7 (n = 5 WT, n = 7 KO), (I) Day 14 (n = 4 WT, n = 4 KO) of CR, and (J) Day 28 (n = 5 WT, n = 7 KO) of CR are shown. (K) The amount of high activity in seconds for *Sirt1*^*loxP/loxP*^ and Nestin-Cre; *Sirt1*^*loxP/loxP*^ mice. On Day 7, the amount of total activity was greater for than *Nestin-Cre; Sirt1*^*loxP/loxP*^ compared to *Sirt1*^*loxP/loxP*^ controls (P = 0.0333). (L) Fraction of high activity measures between *Sirt1*^*loxP/loxP*^ and *Nestin-Cre; Sirt1*^*loxP/loxP*^ groups exhibit a greater amount of FAA in controls on Day 7 (P = 0.0333). For body weight data, statistical significance was determined using an unpaired T test. For behavioral data, statistical significance was determined using the Mann-Whitney Test. * denotes p<0.05.

As we observed little to no effect of selected inactivation of Sirt1 in Pomc-Cre and in pilot studies of tyrosine hydroxylase (TH)-Cre driver [32], which is expressed in dopaminergic, epinephrine, and norepinephrine neurons (S1 Fig), we next sought to create a broader deletion of Sirt1 in the brain. To that end, we utilized the *CamKII-alpha-Cre* [33] to test whether Sirt1 loss in the forebrain would cause a loss of FAA in mice on CR diets. Food intakes between experimental and control groups were similar (P = 0.582, Unpaired T test), with controls averaging 4.5g (+/- 0.7g) and *CamKII-Cre; Sirt1*^*loxP/loxP*^ averaging 4.8g (+/- 1.1g). There were no differences in body weights of *CamKII-Cre; Sirt1*^*loxP/loxP*^ and *Sirt1*^*loxP/loxP*^ littermate controls on CR diets (Fig 2A). High activity waveforms preceding timed CR were similar (Fig 2B), as was the amount of pre-meal high activity at days 14 and 28 of CR (Fig 2C and 2D). There was also no difference in total high activity (Fig 2E) or the fraction of high activity in the three hours before feeding (Fig 2F) between control and experimental groups when on CR across the duration of the study, suggesting that Sirt1 expression in the forebrain was not required for the development or acquisition of FAA.

Because inactivating Sirt1 in bulk of the forebrain had no effect on FAA, our next approach was to create an even broader neuronal knockout by using the *nestin-Cre* driver [34]. We observed a trend toward lower body weights of *nestin-Cre; Sirt1*^*loxP/loxP*^ mice but these differences were not statistically significant (Fig 2G). Food intakes for controls and conditional knockouts were not significantly different (P = 0.168, t test), with controls averaging 4.4g (+/- 0.6g) and knockouts 3.9g (+/- 0.7g). Activity waveforms were similar between groups at Day -7, 14, and 28 with both groups showing a clear increase in activity prior to scheduled mealtime (Fig 2H–2J). The total activity levels (Fig 2K) and FAA levels (Fig 2L) were similar between *nestin-Cre; Sirt1*^*loxP/loxP*^ and *Sirt1*^*loxP/loxP*^ except on Day 7 of CR, when the neuronal knockouts showed a decreased total activity (P = 0.033, Mann-Whitney) and fraction of high activity (P = 0.033, Mann-Whitney). Thus, broad deletion of Sirt1 may cause a slight delay in the acquisition of FAA, but subsequently, there were no significant differences between groups in either total activity or FAA.

**Fig 3:**
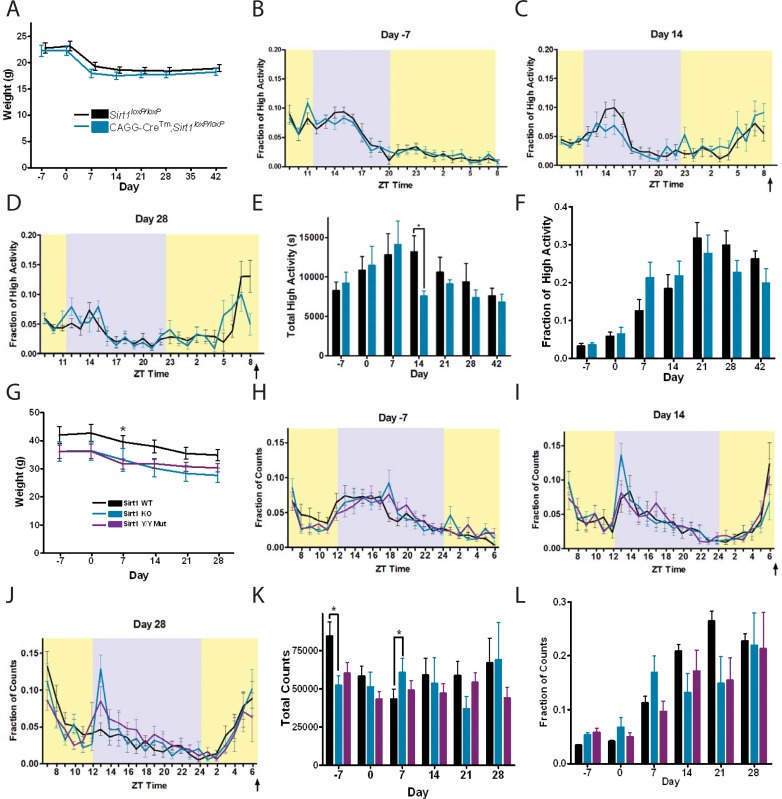
Testing FAA in post-natal knockout, complete null, and catalytic mutant Sirt1 mice. (A) Mean body weight prior to starting CR (Day -7) through Day 42 of CR for tamoxifen-injected *Sirt1*^*loxP/loxP*^ and CAGG-Cre^Tm^; *Sirt1*^*loxP/loxP*^ mice. CR diets began on Day 0. Mean high activity data for home cage behaviors at (B) Day -7 (n = 8 KO, n = 9 WT), (C) Day 14 (n = 9 KO and WT) of CR, and (D) Day 28 (n = 9 KO and WT) of CR are shown. Feeding time is indicated by an arrow. (E) The total high activity for *Sirt1*^*loxP/loxP*^ and CAGG-Cre^Tm^; *Sirt1*^*loxP/loxP*^ groups were similar with the exception of Day 14, where the knockouts showed a significant decrease (n = 9 for both groups, P = 0.0315). (F) Mean normalized high activity in the 3-hours preceding scheduled mealtime were similar between tamoxifen-injected *Sirt1*^*loxP/loxP*^ and CAGG-Cre^Tm^; *Sirt1*^*loxP/loxP*^ mice. (G) Mean body weight for control, Sirt1 KO, and Sirt1 Y/Y mutants starting Day -7, prior to starting CR, through Day 28 of CR. Mean home cage activity counts at (H) Day -7(n = 10 WT, N = 6 KO, and N = 9 YY), (I) Day 14 (n = 10 WT, n = 6 KO, and n = 8 YY) of CR, and (J) Day 28 (n = 10 WT, n = 6 KO, and n = 9 YY) of CR are shown. (K) Total activity counts over the course of the experiment. (I) When dividing the final 3h counts over total counts throughout the 23.5h-24h recording, all mice displayed relatively similar amount of FAA prior to scheduled feeding times on all days except for Day -7 (P = 0.0442). However, no significance was found when performing pairwise comparisons. All data is shown with mean +/- SEM and statistical significance was determined using the Kruskal-Wallis Test with Dunn’s post-test. * denotes p<0.05.

Because a pan-neuronal deletion of using *nestin-Cre* Sirt1 caused only a slight delay in the acquisition of FAA, we next sought a broader deletion of Sirt1 that would also encompass peripheral tissues. Since Sirt1 is important in embryogenesis and its global deletion can cause embryonic lethality [29], we used a ubiquitously active chimeric Cagg promoter to drive tamoxifen-inducible Cre-driver (*CAGG-Cre*^*Tm*^) to globally inactivate Sirt1 during adulthood [35]. We injected *CAGG-Cre*^*Tm*^*; Sirt1*^*loxP/loxP*^ and *Sirt1*^*loxP/loxP*^ mice with tamoxifen at 8–10 weeks of age. Several weeks after tamoxifen injection, we measured food intakes of 4.7g (+/- 1g) for *Sirt1*^*loxP/loxP*^ and 4.5g (+/- of 1.2g) for *CAGG-Cre*^*Tm*^; *Sirt1*^*loxP/loxP*^. The food intakes and body weights (Fig 3A) were not significantly different. High activity waveforms were similar at Day -7 (Fig 3B), and Days 14 and 28 of CR, where a sizable increase in activity before scheduled mealtime was evident (Fig 3C and 3D). The total high activity was also not significantly different between *CAGG-Cre*^*Tm*^*; Sirt1*^*loxP/loxP*^ mice or controls on CR except for Day 14, where controls had significantly greater total activity than *CAGG-Cre*^*Tm*^; *Sirt1*^*loxP/loxP*^ (P = 0.0315, Mann-Whitney, Fig 2E). When we examined high activity in the 3-hours preceding scheduled mealtime, there were no significant differences between controls and postnatal knockouts (Fig 2F), suggesting that global, postnatal deletion of Sirt1 also does not impact FAA behavior.

Finally, we tested both a global, constitutive deletion of Sirt1 [29] and a gene replacement with a catalytically inactive form of Sirt1 in which a histidine at 355 position is changed to a tyrosine, termed “Y/Y”, that is catalytically inactive [36] for FAA. For these studies, we used mice that were middle-aged, ranging from 9 to 11 months of age with average food intakes of 5.5g (+/- 0.87g) for WT, 4.4g (+/- 1.1g) for KO, and 4.8g (+/- 1g) for Y/Y, which were not significantly different (P = 0.1003, T test). There was a trend toward lower body weights in the Sirt1 KO and Y/Y mutants compared to WT; upon CR, all mice showed a substantial and similar decrease in body weight over time (Fig 3G). Particularly, there were no significant differences in weight loss across groups, except for Day 7, when WT showed less weight loss compared to Y/Y (ANOVA, P = 0.0333, Tukey, Fig 3G). For these mice, home cage activity was measured by photobeam breaks and presented as activity “counts” (arbitrary units). The waveforms of normalized activity counts were similar on Day -7, prior to CR (Fig 3H), and upon CR there was a notable increase in activity during ZT4-6, right before mealtime (Fig 3I and 3J). Total activity counts were similar between all groups except for the measurement prior to initiating CR (Day -7) when WT displayed a significantly higher total physical activity than both KO (ANOVA, P = 0.017, Kruskal-Wallis) (Fig 3K). On Day 7, WT mice had significantly lower total counts than Y/Y mutant mice (ANOVA, P = 0.0301, Kruskal-Wallis). However, there were no significant differences between groups on any other day examined (Fig 3K). In terms of pre-meal activity, we found no significant difference between WT, KO, and Y/Y groups on all days of CR (Fig 3L), as observed with all other experimental groups. Thus, all experiments provide little evidence that Sirt1 is key regulator of FAA in mice.

## Discussion

Surprisingly, we observed little evidence that Sirt1 promotes the development of FAA and no evidence for the involvement of Sirt1 in the maintenance of FAA. Nor did we observe much effect on body weight homeostasis during 60% timed CR feeding over a short-term CR experiment—in contrast to well-documented effects of Sirt1 on body weight and metabolism [36, 37]. The *Sirt1*^*loxP/loxP*^ *Pomc-Cre* and *nestin-Cre* mice showed opposite kinetics of FAA development: Pomc deletion accelerated FAA whereas nestin deletion slowed acquisition of FAA. That being said, *CamKII-alpha*, *CAGG-Cre*^*Tm*^, global knockout, and complete gene replacement with catalytically inactive Sirt1 all showed no delay or enhancement in the development of FAA. Thus, we conclude that Sirt1 and the acetylation of the broad network of its substrates are not required for FAA and may only have a very subtle or limited role in circadian entrainment to scheduled feeding. Given the results of Satoh and colleagues [9] demonstrating a strong role for Sirt1 in promoting FAA via hypothalamic pathways, what could account for differences between our study and theirs?

There are several methodological differences between the present study and that of Satoh and colleagues that may account for the discrepancies between our results. To begin with, Satoh and colleagues relied on two genetic strategies to study the function of Sirt1 in mediating the response to dietary restriction. First, they created a transgenic mouse that overexpressed Sirt1 using the ‘half genomic’ prion protein promoter fragment. The Sirt1 transgenic overexpression mice demonstrated enhanced pre-meal activity after 5 days of dietary restriction that correlated with increased c-Fos expression in several hypothalamic nuclei. Their second genetic method relied on a complete deletion mutant of Sirt1 [28] on an FVB background. In contrast, our studies did not utilize a Sirt1 overexpression model and, instead, relied on conditional deletion of exon 4 of Sirt1, a complete knockout using a different targeting strategy [38], and a gene replacement with a catalytically inactive form of Sirt1 [36]. Other potentially important technical differences between studies include the method of activity measurement, diet, and duration of the study. In our studies, we allowed ample time for mice to experience a controlled-feeding schedule—in some cases performing timed CR feeding for 7 weeks (Fig 2)—whereas Satoh and colleagues examined very early development of FAA, displaying data from just the first 5–7 days of dietary restriction. The only effects that we did observe in the conditional mutants occurred during the first week of CR, which is a time of rapid adaptation to extreme weight loss. Perhaps this window of time is when Sirt1 is most important as a mediator of chronic stresses like CR. Finally, Satoh observed stronger effects of Sirt1 deletion in males than in females. While sex differences in FAA are documented, they are subtle [39–42]. As we used both male and female mice in our studies and observed robust inductions of FAA in all groups, where redistribution of activity to precede scheduled meals often exceeded 20% of total activity, we do not support sex differences as an explanation for the discrepancies between our results. To reconcile our results with those of Satoh and colleagues, a more detailed study of the early development and sustainment of FAA conducted by both laboratories using similar activity measurements, time course, and mouse mutants on the same genetic background would be in order.

Sirt1 has a remarkable list of substrates, modulating activity and levels of everything from monoamine oxidase [43] to p53 [44] to c-Myc [45]. The action of Sirt1 in the brain has received much attention in terms of metabolism, feeding-related behaviors, and direct action on the circadian system via hypothalamic neurons [8, 23, 46]. Our results suggest that Sirt1 is not the missing link between scheduled feeding and circadian behavioral activity cycles, but, as has been pointed out several times in the literature, “context is everything” for such a highly-networked protein [38]. In fact, a recent systematic study of the effect of 30 different genetic backgrounds on gene deletion showed profound effects of background on genotype-phenotype relationship in mice [47]. While this study showed that most phenotypes for both diabetes and psychiatric traits were consistent across many strain backgrounds, there were many examples where the trait disappeared or was even reversed on particular backgrounds. Thus, further experiments with conditional deletion of Sirt1 in the brain on different genetic backgrounds may be merited.

## Materials and methods

### Mouse strains and husbandry

The Institutional Animal Care and Use Committee at the California Institute of Technology and the Animal Care and Use Committee at Cal Poly Pomona approved the experiments described herein. The *Sirt1*^*loxP/loxP*^ mice [28] were provided on a genetic C57BL/6 and crossed for one to two generations onto C57BL/6J upon arrival and ovarian transfer rederivation at the Caltech animal facility. A summary of mouse strains used is shown in Table 1. The mutant Sirt1 allele was created on the 129/Sv inbred background and these animals were outcrossed to the outbred CD1 strain. The offspring were intercrossed and mice homozygous for Sirt1 mutations were identified by PCR screening. The Sirt1 Y/Y and KO mice came from the colony of Michael McBurney at the University of Ottawa, while the *Sirt1*^*loxP/loxP*^ mice came from the colony of Lenny Guarente at MIT, and all other mice were obtained from the Jackson Laboratory.

For all experiments, mice were maintained in static microisolator cages, temperatures ranged between 21–23°C, and humidity ranged between 45–65%. The cages contained sani-chip bedding (Envigo, product number 7090) and a cotton nestlet. The conditional mutant mice were fed LabDiet Laboratory Rodent Diet 5001 while the Sirt1 KO and Y/Y mutants were fed 2018 Teklad diet (Envigo). The macronutrient composition of the two diets used in these studies were very similar: the % of calories derived from protein was 28.5% for 5001 chow and 24% for 2018 chow, for fat 13.5% and 18%, and carbohydrates provided 58.0% for both diets. The strong induction of FAA in control mice for all studies (approximately 25% of total activity occurs in the 3-hours preceding scheduled mealtime after 2 weeks of CR, Figs 1I, 2F, 2L, 3F and 3L) suggests that the use of different diets did not influence our results.

The conditional mutant mice were maintained on 13 hours of light and 11 hours of darkness. By convention, for designating time on 13:11 L:D, ZT 12 was designated as the commencement of lights-off. For the Sirt1 knockout and Y/Y mutant (Fig 3), mice were maintained on a 12:12 light:dark cycle, and therefore ZT 0 was defined as lights on, by convention. The strong induction of FAA under both conditions suggests that this minor difference in the amount of daylight did not influence the results of our study.

To genotype mice, DNA was obtained from tail clippings, which were obtained from unanaesthetized 2-week old mice and then digested with proteinase K and DNA was purified using an isopropanol precipitation. For genotyping the Sirt1 genomic locus, we found it essential to use a Qiagen multiplex PCR kit in combination with the following primers: T1 cond KO F GCC CAT TAA AGC AGT ATG TG, T1 cond KO R CAT GTA ATC TCA ACC TTG AG. Cre was amplified with the following primers: GCG GTC TGG CAG TAA AAA CTA TC and GTG AAA CAG CAT TGC TGT CAC TT. Mice were euthanized CO_2_ narcosis.

Tamoxifen (Sigma, T5648) was dissolved in 10% ethanol and sesame oil (Sigma, S3547) at 10 mg/mL. Solutions were prepared the night before and stored at 4°C overnight. Mice were injected intraperitoneally at 8–10 weeks of age with a dose of 10 mg/kg for 5 consecutive days.

### Behavioral measurements and calorie restriction conditions

Home cage behavior measurement began (day -7) at 9–10 weeks of age for mice in Figs 1 and 2. The videos of singly housed mice in the home cage were analyzed by an automated behavior recognition system, HomeCageScan 3.0 [31, 37], and data was output into twenty-four one-hour bins to facilitate the understanding of the temporal structure of activity. Dim red lighting was provided with red LEDs from LEDwholesalers.com (High Power 42 SMT RED LED PAR38). Home cage behavior measurements were obtained by video recording mice from a perpendicular angle in their home cages and analyzing these videos using HomeCageScan software, which annotates for the following behaviors: remain low, pause, twitch, awaken, distance traveled, turn, sniff, groom, food bin entry, chew, drink, stretch, unassigned behaviors, hanging, jumping rearing, and walking. The sum of hanging, jumping, rearing, and walking are designated as high activity behaviors and are summed for sum high activity. FAA was calculated summing the final 3h of high activity and dividing the value by the total high activity in that day per mouse. Because the Sirt1 KO and Y/Y mice had varied coat colors, including white, that are not amenable to HomeCageScan, we used a photo beam system (CLAMS, Columbus Instruments, USA) to quantify homecage activity. Total counts of photo beam breaks that occurred along x- and y- axes as a result of mouse movement, providing bins every 30 minutes. Total activity was determined by summing hanging, walking, jumping and rearing across all 24 hour bins, while FAA ratios were calculated by dividing the high activity in the 3 hours preceding mealtime by the total high activity.

Body weights of mice were weighed every 7^th^ day beginning from day -7 to day 28. Mouse weights on Day -7 reflect ad libitum diets, however, CR diets were employed beginning Day 0. Food intake was calculated for all mice prior to starting CR, while mice were on AL diets. Food intake was measured by placing approximately 50 grams of standard mouse chow in the food bin and measuring remaining chow mass 48 hours later. Daily averages were computed per cohort and 60% of the daily food intake was fed at the same time daily for the duration of the experiment. Feedings occurred at ZT 7 or ZT 9 but were always consistent within an experiment. CR began at 10–11 weeks of age for the conditional mutant mice and 9–11 months of age for the complete knockout and the Y/Y mutant.

### Experimental design and statistical analysis

Statistical significance tests were conducted using GraphPad Instat. Food intakes were tested for significance using an Unpaired T Test, whereas mouse body weights were tested for significance using a One-Way ANOVA with Tukey’s post-test. As behavioral data did not follow a normal distribution, we used nonparametric tests, Mann-Whitney for comparing two groups and Kruskal-Wallis with Dunn’s post-test for comparing three groups.

### Immunohistochemistry and Western blotting

Brain homogenates were prepared in glass dounce homogenizers as 10% homogenates (wt/vol) in PBS. After sonication, large debris was pelleted by low-speed centrifugation. Further dilutions were made into lysis buffer containing (PBS 1% Tween 20 1% Triton X 100 and 150 mM NaCl). The equivalent of 30–50 g of total protein was loaded onto 10% Bis-Tris gels (Invitrogen), transferred to nitrocellulose membranes, and analyzed by Western blotting using the a Sirt1 monoclonal antibody (AS-16, Sigma). Imaging was performed using a Licor Odyssey system.

For immunochemistry, brains were immersion-fixed in 10% buffered formalin (Sigma) for at least 24h prior to being sectioned at room temperature using a vibratome (Leice Instruments). Antibody staining was performed using a rabbit polyclonal Sirt1 antibody (Upstate) and Vector ABC immunoperoxidase staining kit (Vector labs). Images were taken on a Nikon Eclipse TE2000-U light microscope coupled to a computer with NIS-Elements BR 3.0 software.

## Supporting information

S1 Fig Testing FAA in tyrosine hydroxylase Cre deletion mutants of Sirt1. (A) Mean body weights for TH-Cre; *Sirt1*^*loxP/loxP*^ and *Sirt1*^*loxP/loxP*^ littermate controls. CR diets began on Day 0. Mean high activity data for home cage behaviors at (B) Day -7 (n = 5 WT and n = 3 for KO), (C) Day 14 of CR (n = 5 WT and n = 3 KO), and (D) Day 28 (n = 3 WT and n = 2 KO) of CR are shown. (E) Mean total high activity, measured in seconds, over the duration of the experiment. (F) Mean fraction of normalized high activity in the 3h prior to mealtime; there were no differences between groups on all days.
(EPS)

S1 Dataset Behavior, body weight, and food intake for each mouse line used in this study. (XLSX)

## References

[1] Mistlberger RE (1994) Circadian food-anticipatory activity: formal models and physiological mechanisms. *Neurosci Biobehav Rev* 18:171–195 8058212 10.1016/0149-7634(94)90023-x

[2] Davidson AJ (2009) Lesion studies targeting food-anticipatory activity. *Eur J Neurosci* 30:1658–1664 doi: 10.1111/j.1460-9568.2009.06961.x 19863659 10.1111/j.1460-9568.2009.06961.x

[3] Mistlberger RE, Buijs RM, Challet E, Escobar C, Landry GJ, Kalsbeek A, et al (2009a) Standards of evidence in chronobiology: critical review of a report that restoration of Bmal1 expression in the dorsomedial hypothalamus is sufficient to restore circadian food anticipatory rhythms in Bmal1-/- mice. *J Circadian Rhythms* 7:3 19323828 10.1186/1740-3391-7-3 PMC2670815

[4] Mistlberger RE, Buijs RM, Challet E, Escobar C, Landry GJ, Kalsbeek A, et al (2009b) Food anticipation in Bmal1-/- and AAV-Bmal1 rescued mice: a reply to Fuller et al. *J Circadian Rhythms* 7:11 19664274 10.1186/1740-3391-7-11 PMC2734571

[5] Pendergast JS, Nakamura W, Friday RC, Hatanaka F, Takumi T, Yamazaki S (2009) Robust food anticipatory activity in BMAL1-deficient mice. *PLoS One* 4:e4860 doi: 10.1371/journal.pone.0004860 19300505 10.1371/journal.pone.0004860 PMC2654093

[6] Gunapala KM, Gallardo CM, Hsu CT, Steele AD (2011) Single gene deletions of orexin, leptin, neuropeptide Y, and ghrelin do not appreciably alter food anticipatory activity in mice. *PLoS One* 6:e18377 doi: 10.1371/journal.pone.0018377 21464907 10.1371/journal.pone.0018377 PMC3065493

[7] LeSauter J, Hoque N, Weintraub M, Pfaff DW, Silver R (2009) Stomach ghrelin-secreting cells as food-entrainable circadian clocks. *Proc Natl Acad Sci U S A* 106:13582–13587 doi: 10.1073/pnas.0906426106 19633195 10.1073/pnas.0906426106 PMC2726387

[8] Mistlberger RE (2011) Neurobiology of food anticipatory circadian rhythms. *Physiol Behav* 104:535–545 doi: 10.1016/j.physbeh.2011.04.015 21527266 10.1016/j.physbeh.2011.04.015

[9] Satoh A, Brace CS, Ben-Josef G, West T, Wozniak DF, Holtzman DM, et al (2010) Sirt1 promotes the central adaptive response to diet restriction through activation of the dorsomedial and lateral nuclei of the hypothalamus. *J Neurosci* 30:10220–10232 doi: 10.1523/JNEUROSCI.1385-10.2010 20668205 10.1523/JNEUROSCI.1385-10.2010 PMC2922851

[10] Green CB, Takahashi JS, Bass J (2008) The meter of metabolism. *Cell* 134:728–742 doi: 10.1016/j.cell.2008.08.022 18775307 10.1016/j.cell.2008.08.022 PMC3760165

[11] Bass J and Takahashi JS (2010) Circadian integration of metabolism and energetics. *Science* 330:1349–1354 doi: 10.1126/science.1195027 21127246 10.1126/science.1195027 PMC3756146

[12] Ramsey KM, Yoshino J, Brace CS, Abrassart D, Kobayashi Y, Marcheva B, et al (2009) Circadian clock feedback cycle through NAMPT-mediated NAD+ biosynthesis. *Science* 324:651–654 doi: 10.1126/science.1171641 19299583 10.1126/science.1171641 PMC2738420

[13] Asher G, Gatfield D, Stratmann M, Reinke H, Dibner C, Kreppel F, et al (2008) Sirt1 regulates circadian clock gene expression through PER2 deacetylation. *Cell* 134:317–328 doi: 10.1016/j.cell.2008.06.050 18662546 10.1016/j.cell.2008.06.050

[14] Nakahata Y, Kaluzova M, Grimaldi B, Sahar S, Hirayama J, Chen D, et al (2008) The NAD+-dependent deacetylase Sirt1 modulates CLOCK-mediated chromatin remodeling and circadian control. *Cell* 134:329–340 doi: 10.1016/j.cell.2008.07.002 18662547 10.1016/j.cell.2008.07.002 PMC3526943

[15] Noriega LG, Feige JN, Canto C, Yamamoto H, Yu J, Herman MA, et al (2011) CREB and ChREBP oppositely regulate Sirt1 expression in response to energy availability. *EMBO Rep* 12:1069–1076 doi: 10.1038/embor.2011.151 21836635 10.1038/embor.2011.151 PMC3185337

[16] Canto C, Auwerx J (2009a) Caloric restriction, Sirt1 and longevity. *Trends Endocrinol Metab* 20:325–331 19713122 10.1016/j.tem.2009.03.008 PMC3627124

[17] Canto C, Auwerx J (2009b) PGC-1alpha, Sirt1 and AMPK, an energy sensing network that controls energy expenditure. *Curr Opin Lipidol* 20:98–105 19276888 10.1097/MOL.0b013e328328d0a4 PMC3627054

[18] Rodgers JT, Lerin C, Haas W, Gygi SP, Spiegelman BM, Puigserver P (2005) Nutrient control of glucose homeostasis through a complex of PGC-1alpha and Sirt1. *Nature* 434:113–118 doi: 10.1038/nature03354 15744310 10.1038/nature03354

[19] Chalkiadaki A, Guarente L (2012) Sirtuins mediate mammalian metabolic responses to nutrient availability. *Nat Rev Endocrinol* 8:287–296 doi: 10.1038/nrendo.2011.225 22249520 10.1038/nrendo.2011.225

[20] Cakir I, Perello M, Lansari O, Messier NJ, Vaslet CA, Nillni EA (2009) Hypothalamic Sirt1 regulates food intake in a rodent model system. *PLoS One* 4:e8322 doi: 10.1371/journal.pone.0008322 20020036 10.1371/journal.pone.0008322 PMC2790615

[21] Banks AS, Kon N, Knight C, Matsumoto M, Gutierrez-Juarez R, Rossetti L, et al (2008) Sirt1 gain of function increases energy efficiency and prevents diabetes in mice. Cell Metab 8:333–341 doi: 10.1016/j.cmet.2008.08.014 18840364 10.1016/j.cmet.2008.08.014 PMC3222897

[22] Bordone L, Cohen D, Robinson A, Motta MC, van Veen E, Czopik A, et al (2007) Sirt1 transgenic mice show phenotypes resembling calorie restriction. *Aging Cell* 6:759–767 doi: 10.1111/j.1474-9726.2007.00335.x 17877786 10.1111/j.1474-9726.2007.00335.x

[23] Ramadori G, Lee CE, Bookout AL, Lee S, Williams KW, Anderson J, et al (2008) Brain Sirt1: anatomical distribution and regulation by energy availability. *J Neurosci* 28:9989–9996 doi: 10.1523/JNEUROSCI.3257-08.2008 18829956 10.1523/JNEUROSCI.3257-08.2008 PMC2578850

[24] Cohen DE, Supinski AM, Bonkowski MS, Donmez G, Guarente LP (2009) Neuronal Sirt1 regulates endocrine and behavioral responses to calorie restriction. *Genes Dev* 23:2812–2817 doi: 10.1101/gad.1839209 20008932 10.1101/gad.1839209 PMC2800085

[25] Chen D, Steele AD, Lindquist S, Guarente L (2005) Increase in activity during calorie restriction requires Sirt1. *Science* 310:1641 doi: 10.1126/science.1118357 16339438 10.1126/science.1118357

[26] Boily G, Seifert EL, Bevilacqua L, He XH, Sabourin G, Estey C, et al (2008) Sirt1 regulates energy metabolism and response to caloric restriction in mice. *PLoS One* 3:e1759 doi: 10.1371/journal.pone.0001759 18335035 10.1371/journal.pone.0001759 PMC2258149

[27] Prozorovski T, Schulze-Topphoff U, Glumm R, Baumgart J, Schroter F, Ninnemann O, et al (2008) Sirt1 contributes critically to the redox-dependent fate of neural progenitors. *Nat Cell Biol* 10:385–394 doi: 10.1038/ncb1700 18344989 10.1038/ncb1700

[28] Cheng HL, Mostoslavsky R, Saito S, Manis JP, Gu Y, Patel P, et al (2003) Developmental defects and p53 hyperacetylation in Sir2 homolog (Sirt1)-deficient mice. *Proc Natl Acad Sci U S A* 100:10794–10799 doi: 10.1073/pnas.1934713100 12960381 10.1073/pnas.1934713100 PMC196882

[29] McBurney MW, Yang X, Jardine K, Hixon M, Boekelheide K, Webb JR, et al (2003) The mammalian SIR2alpha protein has a role in embryogenesis and gametogenesis. *Mol Cell Biol* 23:38–54 doi: 10.1128/MCB.23.1.38-54.2003 12482959 10.1128/MCB.23.1.38-54.2003 PMC140671

[30] Balthasar N, Coppari R, McMinn J, Liu SM, Lee CE, Tang V, et al (2004) Leptin receptor signaling in POMC neurons is required for normal body weight homeostasis. Neuron 42:983–991. doi: 10.1016/j.neuron.2004.06.004 15207242 10.1016/j.neuron.2004.06.004

[31] Steele AD, Jackson WS, King OD, Lindquist S (2007) The power of automated high-resolution behavior analysis revealed by its application to mouse models of Huntington's and prion diseases. *Proc Natl Acad Sci U S A* 104:1983–1988 doi: 10.1073/pnas.0610779104 17261803 10.1073/pnas.0610779104 PMC1794260

[32] Gong S, Doughty M, Harbaugh CR, Cummins A, Hatten ME, Heintz N, et al (2007) Targeting Cre recombinase to specific neuron populations with bacterial artificial chromosome constructs. *J Neurosci* 27:9817–9823 doi: 10.1523/JNEUROSCI.2707-07.2007 17855595 10.1523/JNEUROSCI.2707-07.2007 PMC6672645

[33] Casanova E, Fehsenfeld S, Mantamadiotis T, Lemberger T, Greiner E, Stewart AF, et al (2001) A CamKIIalpha iCre BAC allows brain-specific gene inactivation. *Genesis* 31:37–42 11668676 10.1002/gene.1078

[34] Tronche F, Kellendonk C, Kretz O, Gass P, Anlag K, Orban PC, et al (1999) Disruption of the glucocorticoid receptor gene in the nervous system results in reduced anxiety. *Nat Genet* 23:99–103 doi: 10.1038/12703 10471508 10.1038/12703

[35] Hayashi S, McMahon AP (2002) Efficient recombination in diverse tissues by a tamoxifen-inducible form of Cre: a tool for temporally regulated gene activation/inactivation in the mouse. *Dev Biol* 244:305–318. doi: 10.1006/dbio.2002.0597 11944939 10.1006/dbio.2002.0597

[36] Seifert EL, Caron AZ, Morin K, Coulombe J, He XH, Jardine K, et al (2012) SirT1 catalytic activity is required for male fertility and metabolic homeostasis in mice. *FASEB J*. 2012 2;26(2):555–66. doi: 10.1096/fj.11-193979 22006156 10.1096/fj.11-193979

[37] Caron AZ, He X, Mottawea W, Seifert EL, Jardine K, Dewar-Darch D, et al (2014) The SIRT1 deacetylase protects mice against the symptoms of metabolic syndrome. *FASEB J*.28(3):1306–16. doi: 10.1096/fj.13-243568 24297700 10.1096/fj.13-243568

[38] McBurney MW, Clark-Knowles KV, Caron AZ, and DA Gray DA (2013) SIRT1 is a Highly Networked Protein That Mediates the Adaptation to Chronic Physiological Stress. *Genes Cancer* 4(3–4):125–34. doi: 10.1177/1947601912474893 24020004 10.1177/1947601912474893 PMC3764470

[39] Hsu CT, Dollar P, Chang D, Steele AD (2010) Daily timed sexual interaction induces moderate anticipatory activity in mice. *PLoS One* 5:e15429 doi: 10.1371/journal.pone.0015429 21082027 10.1371/journal.pone.0015429 PMC2972719

[40] Michalik M, Steele AD, and Mistlberger RE (2015) A sex difference in circadian food-anticipatory rhythms in mice: Interaction with dopamine D1 receptor knockout. *Behav Neurosci*. 129(3):351–60 doi: 10.1037/bne0000058 26030433 10.1037/bne0000058

[41] Li Z, Wang Y, Sunc KK, Wang K, Sun ZS, Zhao M, et al (2015) Sex-related difference in food-anticipatory activity of mice. *Hormones and Behavior* 70: 38–46 doi: 10.1016/j.yhbeh.2015.02.004 25736535 10.1016/j.yhbeh.2015.02.004

[42] Aguayo A, Martin CM, Huddy TF, Ogawa-Okada M, Adkins JL, and Steele AD (2018) Sex differences in circadian food anticipatory activity are not altered by individual manipulations of sex hormones or sex chromosome copy number in mice. PLoS ONE 13(1): e0191373 doi: 10.1371/journal.pone.0191373 29385171 10.1371/journal.pone.0191373 PMC5792018

[43] Libert S, Pointer K, Bell EL, Das A, Cohen DE, Asara JM, et al (2011) Sirt1 activates MAO-A in the brain to mediate anxiety and exploratory drive. *Cell* 147:1459–1472 doi: 10.1016/j.cell.2011.10.054 22169038 10.1016/j.cell.2011.10.054 PMC3443638

[44] Vaziri H, Dessain SK, Ng EE, Imai SI, Frye RA, Pandita TK, et al (2001) hSIR2(Sirt1) functions as an NAD-dependent p53 deacetylase. *Cell* 107:149–159 11672523 10.1016/s0092-8674(01)00527-x

[45] Menssen A, Hydbring P, Kapelle K, Vervoorts J, Diebold J, Luscher B, et al (2012) The c-Myc oncoprotein, the NAMPT enzyme, the SIRT1-inhibitor DBC1, and the SIRT1 deacetylase form a positive feedback loop. *Proc Natl Acad Sci* 109(4):E187–E196 doi: 10.1073/pnas.1105304109 22190494 10.1073/pnas.1105304109 PMC3268300

[46] Orozco-Solis R, Ramadori G, Coppari R, and and Sassone-Corsi P (2015) SIRT1 Relays Nutritional Inputs to the Circadian Clock Through the Sf1 Neurons of the Ventromedial Hypothalamus. *Endocrinology* 156(6): 2174–2184. doi: 10.1210/en.2014-1805 25763637 10.1210/en.2014-1805 PMC4430615

[47] Sittig LJ, Carbonetto P, Engel KA, Krauss KS, Barrios-Camacho CM, and Palmer AA (2016) Background Limits Generalizability of Genotype-Phenotype Relationships. *Neuron* 91(6): 1253–1259 doi: 10.1016/j.neuron.2016.08.013 27618673 10.1016/j.neuron.2016.08.013 PMC5033712

